# The effect of age on short-term and mid-term outcomes after thoracoscopic Ivor Lewis esophagectomy: a propensity score-matched analysis

**DOI:** 10.1186/s12893-021-01435-5

**Published:** 2021-12-20

**Authors:** Friederike Martin, Dino Kröll, Sebastian Knitter, Tobias Hofmann, Jonas Raakow, Christian Denecke, Johann Pratschke, Matthias Biebl

**Affiliations:** Department of Surgery, Campus Charité Mitte | Campus Virchow Klinikum, Charité – Universitätsmedizin Berlin, corporate member of Freie Universität Berlin, Humboldt-Universität Zu Berlin and Berlin Institute of Health, Augustenburger Platz 1, 13353 Berlin, Germany

**Keywords:** Outcome, Age, Thoracoscopic esophagectomy, Ivor Lewis, Esophageal cancer

## Abstract

**Background:**

The number of elderly patients diagnosed with esophageal cancer rises. Current information about outcomes in elderly patients undergoing thoracoscopic Ivor Lewis esophagectomy is limited. The objective of this study was to evaluate the influence of age on short-and mid-term outcomes after thoracoscopic Ivor Lewis esophagectomy.

**Methods:**

A retrospective review of 188 patients with esophageal cancer undergoing thoracoscopic Ivor Lewis esophagectomy between August 2014 and July 2019 was performed. Patients were divided into patients aged > 75 years (elderly group (EG), n = 37) and patients ≤ 75 years (younger group (YG), n = 151) and matched using propensity-score matching. Baseline characteristics, length of hospital stay, mortality and major postoperative complications (Clavien-Dindo ≥ grade III) were compared.

**Results:**

After matching 74 patients remained (n = 37 in each group). Postoperatively, no significant differences in major and overall complications, intra-hospital and 30-day mortality, disease-free or overall survival up to 3 years after surgery were noted. The incidence of pulmonary complications (65% vs. 38%) and pneumonia (54% vs. 30%) was significantly higher and the median hospital length of stay (12 vs. 14 days) significantly longer in the EG versus YG.

**Conclusion:**

Thoracoscopic Ivor Lewis esophagectomies resulted in acceptable postoperative major morbidity and mortality without compromising 3-years overall and disease-free survival in elderly compared to younger patients with esophageal cancer. However, the incidence of postoperative pulmonary complications was higher in patients aged over 75 years.

**Supplementary Information:**

The online version contains supplementary material available at 10.1186/s12893-021-01435-5.

## Introduction

Esophageal cancer (EC) is the ninth most common cancer in adults worldwide, with an increased adenocarcinoma incidence [1]. Despite multimodal therapy concepts, the prognosis for patients with EC is still abysmal [2]. Due to demographic developments, the population will become increasingly older in the coming decades. Thus, the number of advanced aged patients (> 75 years) with EC will also increase [3]. Elderly patients represent a special group of patients due to a higher incidence of comorbidities and fragility compared to younger patients [4]. This poses a particular challenge for therapy decisions in elderly patients with esophageal cancer. Therapeutic options for EC usually include surgery, chemotherapy, and radio (chemo) therapy, depending on the tumors’ histological type, tumor stage, and the individual patient’s comorbidities [5].

Esophageal resection remains the treatment of choice after neoadjuvant treatment for advanced resectable esophageal tumors. Despite the enormous improvement of surgical techniques in recent years, esophagectomy remains a challenging procedure with a high risk for postoperative morbidity and mortality in both younger and older patients [6].

Literature also discusses the subject of whether age influences the postoperative outcome [7–9] controversial, especially after minimally invasive Ivor Lewis esophagectomy (MIE) [10], since MIE has been shown to reduce pulmonary complications and hospital length of stay compared to open esophagectomy without compromising oncologic safety [6, 11]. The influence of age on postoperative morbidity, especially in patients undergoing minimally invasive esophagectomy, is little observed. All studies were retrospective without longer follow-up, patients older than 75 years were excluded in the recent essential clinical trials [11, 12], and none of the studies were corrected for case-mix parameters. Therefore, the present study aims to compare postoperative morbidity and survival over three years in patients undergoing thoracoscopic Ivor Lewis esophagectomy with distal esophageal or esophagogastric junction carcinoma with case-mix correction by propensity score-matching analysis.

## Methods

Retrospective data analysis was performed, including prospectively collected data of 188 patients undergoing thoracoscopic Ivor Lewis esophagectomy in our clinic between August 2014 and July 2019. According to their age, patients were categorized into ≤ 75 years (EG, elderly group; n = 37) and > 75 years (YG, younger group, n = 151).

Patients 18 to 88 years old with resectable EC (cT1–4a N0–3 M0) of the intrathoracic esophagus or esophagogastric junction (Siewert type I and Siewert type I–II) treated with totally minimally invasive, robotic or hybrid (abdomen open, thorax laparoscopic) esophagectomy were eligible for inclusion. Anastomotic techniques, according to the Ivor Lewis technique, were mechanical circular end-to-side anastomosis. Pyloric drainage procedures were not routinely performed. Adenocarcinomas and squamous cell carcinomas were included. Patients with benign diseases and all patients with cancer of the gastric cardia were excluded. Curative resection after neoadjuvant [(radio) chemotherapy] treatment was the standard of care. The study was approved by the institutional ethics committee.

### Outcome measures and definitions

Patient demographics, details regarding the surgical procedure, neoadjuvant chemoradiotherapy (nCRT), tumor-specific variables, and survival outcomes were recorded. A routine pathology workup was performed as recommended [13]. Tumors were classified according to the World Health Organization classification [14], and staging was performed according to the UICC/American Joint Committee on Cancer (eighth edition) criteria [15].

The primary endpoint was major postoperative complication as a surgical complication with the Clavien-Dindo classification grade III or higher [16]. Postoperative (overall and minor) complications included anastomotic leakage, respiratory complications, according to the ECCG guidelines [17], pneumonia, cardiovascular complications, wound infections, and other complications (i.e. anastomotic stricture). Postoperative all-cause mortality (in-hospital and 30-day mortality) was noted. Long-term follow-up data were collected by chart review and, in case of missing data, by contacting the general practitioner or the patient directly.

### Statistical analysis

All patients were stratified according to their age as described and propensity scores were then used to match patients ≤ 75 years at resection with those > 75 years of age at resection. A 1:1 propensity-score matching based on logistic regression with a match tolerance of 0.1 was performed based on the following matching parameters: Sex, BMI, American Society of Anesthesiologists classification, comorbidities, tumor type, clinical stage and neoadjuvant treatment. Quantitative and qualitative variables were expressed as medians (IQR or range) and frequencies. Categorical and continuous variables were compared between YG and EG using the Chi-square, Fisher’s exact, or Mann–Whitney *U* test as appropriate. Overall survival (OS) was calculated from the date of resection to the date of death or last follow-up and disease-free survival (DFS) was calculated from the date of resection to the date of diagnosis of recurrent disease or last follow-up. Log-rank tests were than used to compare OS and DFS between YG and EG. p values < 0.05 were considered statistically significant. SPSS software package, version 25, by IBM (Armonk, NY) was used for statistical analyses.

## Results

### Baseline characteristics

Data from 188 consecutive patients who underwent thoracoscopic Ivor Lewis esophagectomy between 2014 and 2019 were analyzed. Before matching, significant differences between the two groups were present for age at resection (63 vs. 78 years, *p* < 0.0001), cardiovascular diseases (60% vs. 78%, *p* = 0.034), renal insufficiency (7% vs. 19%, *p* = 0.048), and use of neoadjuvant chemotherapy (91% vs. 73%, *p* = 0.005) (Table 1).

**Table 1:** Comparison of case-mix characteristics in the overall population and in patients younger than 75 years (younger group) and older than 75 years (elderly group) undergoing thoracoscopic ivor lewis esophagectomy before and after propensity score matching

Characteristics	All patients (n = 188)	Before matching			After matching		
		YG (n = 151)	EG (n = 37)	*p*	YG (n = 37)	EG (n = 37)	*p*
Median age, years (IQR)	65.5 (58–74)	63 (56–69)	78 (77–80.5)	** < 0.0001**	63 (55.5–68.5)	78 (77–80.5)	** < 0.0001**
Sex, n (%)				0.268			1
Female	43 (23)	32 (21)	11 (30)		11 (30)	11 (30)	
Male	145 (77)	119 (79)	26 (70)		26 (70)	26 (70)	
Median BMI, kg/m^2^ (IQR)	26 (23.1–29)	26 (23–29)	26 (24–29)	0.736	26 (22–28.85)	26 (24–29)	0.565
Diabetes, n (%)	30 (16)	22 (15)	8 (22)	0.294	5 (14)	8 (22)	0.359
Cardiovascular disease, n (%)	119 (63)	90 (60)	29 (78)	**0.034**	27 (73)	29 (78)	0.588
Pulmonary disease, n (%)	38 (20)	31 (21)	7 (19)	0.827	8 (22)	7 (19)	0.772
Renal insufficiency, n (%)	17 (9)	10 (7)	7 (19)	**0.048**	7 (19)	7 (19)	1
Liver cirrhosis, n (%)					0 (0)	1 (3)	1
ASA physical status, n (%)				0.528			0.561
ASA I	5 (3)	5 (3)	0 (0)		1 (3)	0 (0)	
ASA II	67 (37)	55 (39)	12 (32)		14 (39)	12 (32)	
ASA III	105 (58)	81 (57)	24 (65)		19 (53)	24 (65)	
ASA IV	3 (2)	2 (1)	1 (3)		2 (5)	1 (3)	
Tumor location, n (%)				0.182			0.159
Esophagus	103 (55)	79 (53)	24 (65)		18 (49)	24 (65)	
Gastroesophageal junction	84 (45)	71 (47)	13 (35)		19 (51)	13 (35)	
Preoperative chemotherapy, n (%)	165 (88)	138 (91)	27 (73)	**0.005**	29 (78)	27 (73)	0.588
Preoperative radiotherapy, n (%)	68 (36)	59 (39)	9 (24)	0.089	9 (24)	9 (24)	1
T category, n (%)				0.929			0.487
T1	12 (7)	10 (7)	2 (5)		6 (17)	2 (5)	
T2	21 (12)	17 (12)	4 (11)		4 (11)	4 (11)	
T3	136 (76)	108 (76)	28 (76)		25 (69)	28 (76)	
T4	10 (6)	7 (5)	3 (8)		1 (3)	3 (8)	
N category, n (%)				0.381			0.829
N0	56 (31)	47 (33)	9 (24)		11 (31)	9 (24)	
N1	54 (30)	41 (29)	13 (35)		11 (31)	13 (35)	
N2	45 (25)	33 (23)	12 (32)		9 (26)	12 (32)	
N3	24 (13)	21 (15)	3 (8)		4 (12)	3 (8)	
Histologic type, n (%)				0.618			0.634
Adenocarcinoma	126 (70)	99 (69)	27 (73)		29 (78)	27 (73)	
Squamous cell carcinoma	55 (30)	45 (31)	10 (27)		8 (22)	10 (27)	
UICC stage, n (%)				0.712			0.229
I	17 (10)	15 (11)	2 (5)		5 (14)	2 (5)	
II	42 (24)	33 (23)	9 (24)		8 (23)	9 (24)	
III	109 (61)	86 (61)	23 (62)		22 (63)	23 (62)	
IV	10 (6)	7 (5)	3 (8)		0 (0)	3 (8)	

*P* < 0.05 was considered statistically significant and highlighted by bold letters

*IQR* interquartile range; *BMI* body-mass index; *ASA* American Society of Anesthesiologists; *UICC* Union for International Cancer Control

After matching, 74 patients remained to be evaluated (37 in each group). As expected, median age of resection remained significantly different between the EG and YG group (63 vs. 78 years, *p* < 0.0001), all other preoperative parameters were not significantly different. Details of characteristics before and after propensity score matching are demonstrated in Table 1.

### Postoperative complications

Details of outcome parameters before and after matching are shown in Table 2. After matching, the rate of postoperative pneumonia was 30% and 54% (p = 0.034), and pulmonary complications, were 46% and 65% in the YG in the EG (p = 0.020), respectively. Those differences were also present when excluding patients who underwent hybrid or robotic Ivor Lewis esophagectomy (data shown in a supplementary Additional file 1: Table S1). There were no significant differences in major (35% vs. 57%, p = 0.062) and overall complications (69% vs. 78%, p = 0.422). Postoperative in-hospital mortality as well as 30-day mortality was not significantly different between the groups.

**Table 2:** Comparison of outcome parameters between patients younger than 75 years (younger group) and older than 75 years (elderly group)

Characteristics	All patients (n = 188)	Before matching			After matching		
		YG (n = 151)	EG (n = 37)	*p*	YG (n = 37)	EG (n = 37)	*p*
Median number of lymph nodes removed (IQR)	30 (23.8–38)	30 (24–37)	31 (19–38)	0.649	32 (25–36.5)	31 (19–38)	0.511
Positive resection margins, n (%)	15 (8)	12 (8)	3 (8)	1	2 (5)	3 (8)	0.674
Type of resection, n (%)				0.935			0.476
MIE	129 (69)	104 (69)	25 (68)		23 (62)	25 (68)	
Hybrid (abdominal)	27 (14)	21 (14)	6 (16)		4 (11)	6 (16)	
Robotic	32 (17)	26 (17)	6 (16)		10 (27)	6 (16)	
Overall morbidity, n (%)	136 (73)	108 (72)	28 (78)	0.483	25 (69)	28 (78)	0.422
Major postoperative morbidity, n (%)	96 (51)	75 (50)	21 (57)	0.440	13 (35)	21 (57)	0.062
Anastomotic leak, n (%)	29 (15)	22 (15)	7 (19)	0.512	7 (19)	7 (19)	1
Anastomotic stricture, n (%)	11 (6)	10 (7)	1 (3)	0.695	3 (8)	1 (3)	0.615
Pulmonary complications, n (%)	94 (50)	70 (46)	24 (65)	**0.044**	14 (38)	24 (65)	**0.020**
Postoperative pneumonia, n (%)	67 (36)	47 (31)	20 (54)	**0.009**	11 (30)	20 (54)	**0.034**
Median duration of hospital stay (IQR), days	15 (12–30.8)	15 (12–30)	21 (14.5–33)	**0.025**	14 (12–29.5)	21 (14.5–33)	0.050
In-hospital mortality, n (%)	6 (3)	4 (3)	2 (5)	0.337	1 (3)	2 (5)	1
30-day mortality, n (%)	2 (1)	1 (1)	1 (3)	0.357	0 (0)	1 (3)	1

*P* < 0.05 was considered statistically significant and highlighted by bold letters

*MIE* minimally invasive esophagectomy

### Other outcome parameters

**Fig. 1:**
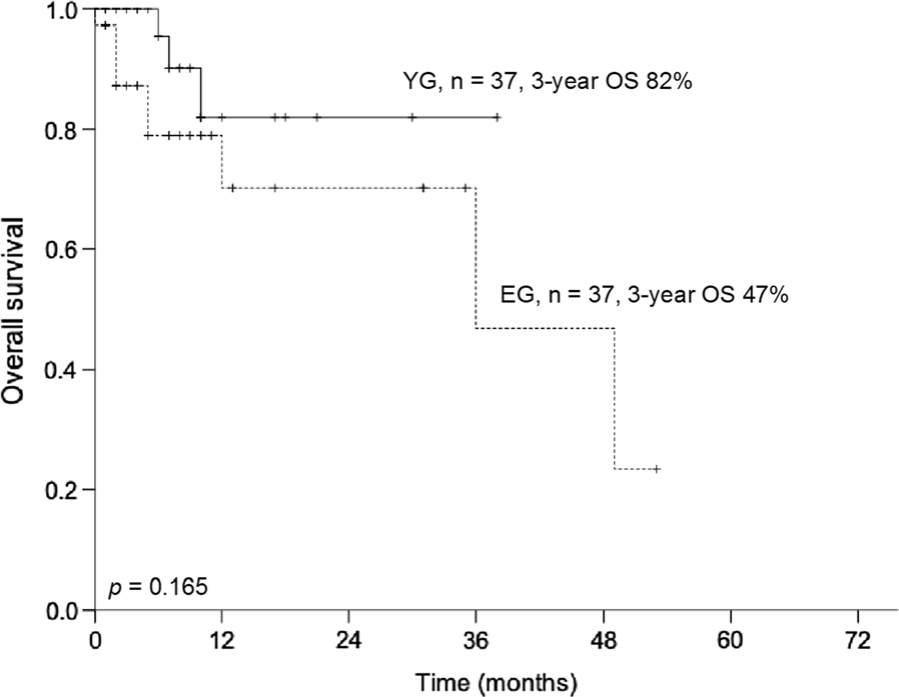
Three-year overall survival between propensity-score matched patients younger than 75 years (younger group) and older than 75 years (elderly group)

**Fig. 2:**
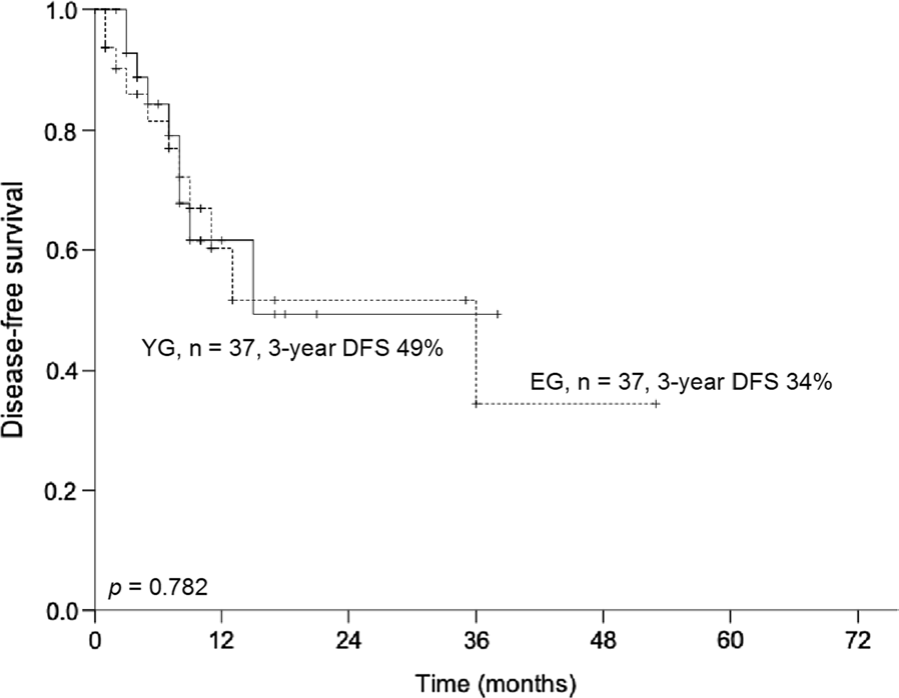
Three-year disease-free survival between propensity-score matched patients younger than 75 years (younger group) and older than 75 years (elderly group)

The median hospital length of stay was 14 days in the YG and 21 days in the EG (p = 0.050). The median number of examined lymph nodes was 32 in the YG and 31 in the EG (p = 0.511). Three-years OS was 82% in the YG and 47% in the EG (p = 0.165; Fig. 1). Three-years DFS was 49% in the YG and 34% in the EG (p = 0.782; Fig. 2). All other outcome parameters were not significantly different between the groups (Table 2).

## Discussion

In this propensity score-matched single-center cohort study, no significant differences were seen in overall and major postoperative complications and mortality after thoracoscopic Ivor Lewis esophagectomy comparing elderly and younger patients. Besides, there were no significant differences in long-term survival. The widespread belief that age harms major complications and long-term outcomes is not in line with the present study's findings, and age alone is not a contraindication for MIE [9].

However, the incidence of pulmonary complications and the rate of postoperative pneumonia was significantly higher and hospital length of stay was significantly longer in the elderly group.

Despite recent advantages in perioperative care and minimally invasive surgical techniques, the risk of pulmonary complications after esophagectomy is relevant [18, 19]. Compared to open esophagectomy, elderly patients may benefit from MIE. Mariette et al. and Biere et al. demonstrated in their trials that the incidence of pneumonia was lower (50–70%) after MIE compared to open esophagectomy [6, 11]. Nevertheless, our results indicate that age is associated with a higher risk for pulmonary complications after MIE, which would support other studies in which age is described as an independent risk factor for pulmonary complications [20]. Furthermore, it was shown that sarcopenia, which occurred more often in elderly patients, was associated with increased rates of pulmonary complications after esophagectomy [21, 22].

An important risk factor for the development of postoperative complications are preoperative comorbidities and the thorough assessment of elderly patients is essential. While chronological age per se has proven to be not predictive for operative success in many major abdominal surgeries, frailty irrespective of age has proven to be associated with higher rates of mortality, postoperative complications, length of stay in older surgical patients. The multimodal assessment and interventions and assessment in the form of preoperative (respiratory) prehabilitation are warranted in order to improve the outcome in elderly high-risk patients [10, 23–25].

While we and others have found that postoperative hospital stay after MIE was increased in elderly patients, we however would argue that this parameter is not clinically utmost relevant [10]. Instead, an evaluation of true return to preoperative level of function after surgery might be a more important factor to compare outcomes after major cancer surgery in frail patients. Due to the study's retrospective nature, the exact reasons for the herein observed prolonged hospital stay of elderly patients after MIE are speculative.

Interestingly, both in the unmatched and in the matched cohort, surgical complications such as anastomotic leaks, postoperative hemorrhage and reoperation rates did not occur in higher percentages in the elderly cohort.

An important limitation in this retrospective cohort is the relatively small number of elderly patients and the fact that we had not defined specified criteria for selecting elderly patients for MIE preoperatively.

However, to lower the risk of selection bias, a propensity-score matched analysis was performed and the American Society of Anesthesiologists physical status and the comorbidities did not differ between the groups. Selection bias may still remain as unknown or unrecorded covariates may have influenced the matching process. Still, we are confident in our results as all registered baseline parameters were equivalent between the groups after matching.

Furthermore, compared to patients with non-surgical treatment (e.g., unfit patients, toxicity side effects of neoadjuvant radiochemotherapy) would be exciting in the future, e.g., comparing the long-term prognosis but was unfortunately not possible to analyze out of the surgical database.

## Conclusions

Thoracoscopic Ivor Lewis esophagectomy in elderly patients aged above 75 years is associated with a comparable incidence of major and overall complications, 30-day mortality, and mid-term survival compared to patients younger than 75. However, the incidence of pulmonary complications and pneumonia is lower in younger patients. Intensive respiratory prehabilitation may be beneficial in elderly patients undergoing Thoracoscopic Ivor Lewis esophagectomy.

## Supplementary Information

**Additional file 1: Table S1.** Subgroup analysis of outcome parameters between patients younger than 75 years (younger group) and older than 75 years (elderly group) who underwent totally MIE.

## Data Availability

The authors confirm that the analyzed data supporting the findings of this study are available within the article. The raw data are available on request from the corresponding author.

## Abbreviations

ASA: American Society of Anesthesiologists
BMI: Body-mass-index
DFS: Disease free survival
EC: Esophageal cancer
EG: Elderly group
IQR: Interquartile range
MIE: Minimally invasive esophagectomy
nCRT: Neoadjuvant chemoradiotherapy
OS: Overall survival
UICC: Union internationale contre le cancer
YG: Younger group
