# Supplementary material for: The effect of age on short-term and mid-term outcomes after thoracoscopic Ivor Lewis esophagectomy: a propensity score-matched analysis
Source: BMC Surg. 2021 Dec 20;21:431. doi: 10.1186/s12893-021-01435-5 (PMC8686649; doi:10.1186/s12893-021-01435-5)
Supplement: Supplementary file 1 — Additional file 1: Table S1. Subgroup analysis of outcome parameters between patients younger than 75 years (younger group) and older than 75 years (elderly group) who underwent totally MIE. [file 12893_2021_1435_MOESM1_ESM.docx]

**Supplementary Table 1.** Subgroup Analysis of Outcome Parameters Between Patients Younger than 75 Years (Younger Group) and Older than 75 Years (Elderly Group) who underwent totally MIE

| **Characteristics** | **YG**  **(n = 23)** | **EG**  **(n = 25)** | ***p*** |
| --- | --- | --- | --- |
|  |  |  |  |
| Median number of lymph nodes removed (IQR) | 30 (15-60) | 29 (7-50) | 0.321 |
| Positive resection margins, n (%) | 1 (4) | 1 (4) | 1 |
| Overall morbidity, n (%) | 16 (73) | 21 (84) | 0.480 |
| Major postoperative morbidity, n (%) | 11 (48) | 16 (64) | 0.259 |
| Anastomotic leak, n (%) | 7 (30) | 5 (20) | 0.404 |
| Anastomotic stricture, n (%) | 3 (13) | 1 (4) | 0.338 |
| Pulmonary complications, n (%) | 11 (48) | 19 (76) | **0.044** |
| Postoperative pneumonia, n (%) | 9 (39) | 17 (68) | **0.045** |
| Median duration of hospital stay (IQR), days | 23 (9-239) | 20 (12-153) | 0.672 |
| 30-day mortality, n (%) | 0 (0) | 1 (4) | 1 |
| 90-day mortality, n (%) | 0 (0) | 3 (16) | 0.231 |
| In-hospital mortality, n (%) | 1 (4) | 2 (8) | 1 |

MIE, minimally invasive esophagectomy; IQR, interquartile range.
